# Jottings

**Published:** 1910-01-15

**Authors:** 


### Jottings.

On December 10 last a severe earthquake shock wrecked the Women's and Children's Hospital at Guam.

The Bournemouth Guardians have decided to erect a new infirmary at the Christchurch Workhouse, which will involve an expenditure of about £11,000.

The Six Thousand Shilling Fund instituted by the Surrey Court on behalf of the Teddington Hospital has been a great success. A total of over £370 has been subscribed.

An entertainment and an excellent concert were given this week at the Metropolitan Hospital in Kingsland Road, when many friends of the patients and subscribers to the funds visited the institution.

The Acton Council has accepted a tender for the erection of a new pavilion, to contain thirty beds, and application has been made to the Local Government Board for sanction to a loan of £4,650, to cover cost of building and furniture.

The annual entertainment at the Alexandra Hospital for Children with Hip Disease took place on January 5, and a special fund having been raised for the purpose, the fun and good fellowship was not a charge upon the hospital's finances.

The late Mr. Edward Malam, of Hanley, a brewer, has left about £35,000 upon trust to purchase a site and to erect thereon a convalescent home to be called by his name, the endowment of the home being for the benefit of his native town of Tunstall.

The Board of the Anti-Vivisection Hospital, Battersea, held a meeting at the hospital last week to consider the statements recently made by Coroner Troutbeck, of which a full report appeared in The Hospital for January 8, concerning the treatment of patients. A statement was prepared, and will be issued to the newspapers.

The Wood Green Urban District Council decided on December 23 to make an annual grant of £120 towards the maintenance of the Wood Green, Hornsey, and Southgate Hospital. The adjoining district of Southgate has, through its council, also decided to help by contributing £1 per in-patient from the Southgate district.

Christmas festivities at the Chelsea Hospital for Women ended last Saturday with an entertainment arranged by the Ladies' Committee. Last year 803 patients were admitted and 425 critical operations performed. The mortality rate is but one and a half. The convalescent home at St. Leonard's has been most fruitful in results.

The annual dinner of past and present students of the Royal London (Moorfields) Ophthalmic Hospital will be held at the Trocadéro Restaurant on Wednesday, January 26, under the presidency of Mr. Edward Nettleship, consulting surgeon to the hospital. Tickets, price 10s. 6d., exclusive of wine, may be obtained from the Hon. Secretaries, Mr. Arnold Lawson, 12 Harley Street, W., and Mr. J. Herbert Parsons, 54 Queen Anne Street, W.

